# QTL Mapping of Genome Regions Controlling Temephos Resistance in Larvae of the Mosquito *Aedes aegypti*

**DOI:** 10.1371/journal.pntd.0003177

**Published:** 2014-10-16

**Authors:** Guadalupe del Carmen Reyes-Solis, Karla Saavedra-Rodriguez, Adriana Flores Suarez, William C. Black

**Affiliations:** 1 Laboratorio de Arbovirología, Centro de Investigaciones Regionales Dr. Hideyo Noguchi, Universidad Autónoma de Yucatán, Mérida, Yucatán, México; 2 Department of Microbiology, Immunology and Pathology, Colorado State University, Fort Collins, Colorado, United States of America; 3 Laboratorio de Entomología Médica, Facultad de Ciencias Biológicas, Universidad Autónoma de Nuevo León, San Nicolás de los Garza, Nuevo León, México; University of California, Irvine, United States of America

## Abstract

**Introduction:**

The mosquito *Aedes aegypti* is the principal vector of dengue and yellow fever flaviviruses. Temephos is an organophosphate insecticide used globally to suppress *Ae. aegypti* larval populations but resistance has evolved in many locations.

**Methodology/Principal Findings:**

Quantitative Trait Loci (QTL) controlling temephos survival in *Ae. aegypti* larvae were mapped in a pair of F_3_ advanced intercross lines arising from temephos resistant parents from Solidaridad, México and temephos susceptible parents from Iquitos, Peru. Two sets of 200 F_3_ larvae were exposed to a discriminating dose of temephos and then dead larvae were collected and preserved for DNA isolation every two hours up to 16 hours. Larvae surviving longer than 16 hours were considered resistant. For QTL mapping, single nucleotide polymorphisms (SNPs) were identified at 23 single copy genes and 26 microsatellite loci of known physical positions in the *Ae. aegypti* genome. In both reciprocal crosses, Multiple Interval Mapping identified eleven QTL associated with time until death. In the Solidaridad×Iquitos (SLD×Iq) cross twelve were associated with survival but in the reciprocal IqxSLD cross, only six QTL were survival associated. Polymorphisms at acetylcholine esterase (AchE) loci 1 and 2 were not associated with either resistance phenotype suggesting that target site insensitivity is not an organophosphate resistance mechanism in this region of México.

**Conclusions/Significance:**

Temephos resistance is under the control of many metabolic genes of small effect and dispersed throughout the *Ae. aegypti* genome.

## Author Summary

The mosquito *Aedes aegypti* is the principal vector of dengue and yellow fever flaviviruses. Due to a lack of effective drugs or vaccines, if an epidemic of dengue fever occurs in the near future, the first line of defense will involve the use of insecticides to suppress adult populations of *Ae. aegypti*. Unfortunately, the species has become resistant to most of the insecticides that can be safely applied. The authors have worked extensively on the mechanisms of resistance to the various insecticides commonly used for suppression of *Ae. aegypti* populations. Temephos is an organophosphate insecticide used globally to suppress *Ae. aegypti* larval populations but resistance has evolved in many locations. In this study we show that temephos resistance is under the control of many metabolic genes of small effect and dispersed throughout the *Ae. aegypti* genome. This information will be of general interest to field workers involved in the suppression of field populations of *Ae. aegypti*.

## Introduction

*Aedes aegypti* is the principal vector of Dengue Fever (DENV) and Yellow Fever (YFV) flaviviruses throughout tropical and subtropical regions of the world and 2.5 billion people are at risk for DENV infection [1]. Currently DENV vaccines have low efficacy [2], [3] so that vector control remains the only option to reduce or prevent DENV transmission. Adult control depends largely on the use of pyrethroid insecticides. However, resistance to pyrethroids has been rising globally [4], [5], [6], [7], [8], [9]. More sustained control can potentially be achieved through the placement of insecticides in water containers that are known to harbor developing *Ae. aegypti* larvae in and around human habitations. For larval control, the three most widely used compounds are *Bacillus thuringiensis israelensis* (*Bti*), methoprene, and temephos. Globally, temephos is the most widely used of these three due to its very low vertebrate toxicity, relatively low cost, the fact that methoprene is a growth regulator with greatest effectiveness against older (third and fourth instar) larvae [10] and, because *Bti* must be ingested to be effective, it does not affect late larval or pupal stages when active feeding has ceased. Temephos is one of a few organophosphates registered to control *Ae. aegypti* larvae, and is the only organophosphate with any appreciable larvicidal use.

Temephos was first registered in the United States for mosquito control in 1965. It was quickly adopted as a larvicide because it was effective in polluted water, had a long residual activity, was available in several use-specific formulations, had a different mode of action than alternatives, and could be used on any larval instar. Temephos is toxic to many mosquito vector species that grow in a diversity of stagnant, saline, brackish and temporary water bodies. It remains an important management tool for mosquito abatement programs. The most widely used commercial preparation of temephos is Abate (EPA Registration No. 8329-60, Clarke Mosquito Control Products, Inc., Roselle, IL).

Temephos was used for 30 years before initial reports of resistance appeared in 1995. Initial studies reported less than a 5-fold resistance ratio (RR) in *Ae. aegypti* collections from Falcon and Aragua states of Venezuela [11]. In 1995, larvae from 34 strains of *Ae. aegypti* from 17 Caribbean countries were bioassayed and there were fairly high levels of temephos resistance in Tortola, British Virgin Islands (RR = 10–12) and Antigua (RR = 6–9) [12]. In 1999 a Tortola collection of *Ae. aegypti* was tested and a RR = 47 was identified [13]. After 13 generations of temephos laboratory selection, the RR increased to 181 fold [13]. Since 2000, temephos resistance has been reported from Cuba and Venezuela [14], [15], Thailand [16], the Brazilian states of Sao Paulo [17], Espirito Santo, Rio de Janeiro [18], Sergipe, Alagoas, [19], Ceara [20], and Paraiba [21]. Most recently reports have appeared from El Salvador [22], Martinique Island in the French West Indies [23], Argentina [24], [25], India [26], Colombia [27], and Trinidad [28], [29]. Although resistance to temephos has been demonstrated in many areas of the world, it is the only remaining organophosphate larvicide with any appreciable use. As such, it is an important tool in resistance management programs that depend on alternative larvicides. Alteration in the registration status or availability of temephos would have a large negative impact on our ability to control DENV transmission globally.

The purpose of the present study was to develop a better understanding of the genetics underlying temephos resistance in *Ae. aegypti* using QTL mapping in recently collected strains. A strain previously established from Solidaridad, Mexico was selected to have 290 fold higher temephos resistance than another strain that had been established from Iquitos, Peru. Parents from these two strains were reciprocally crossed to generate F_1_ siblings which were then intercrossed to generate an F_2_. The F_2_ generations were not large enough to assay for temephos resistance and so an F_3_ was generated through additional sib mating. F_3_ larvae were exposed to a discriminating dose of temephos and then checked every two hours up to 16 hours. Dead mosquitoes were preserved for DNA isolation at each time point and those surviving longer than 16 hours were considered resistant.

## Methods

### Aedes aegypti strains

Two strains of *Aedes aegypti* were used. A F_3_ strain collected from Iquitos, Perú was kindly provided by Dr. Amy Morrison (University of California, Davis). A second strain raised during two generations in the lab was collected by the authors from the neighborhood of Solidaridad, in the city of Chetumal, in the state of Quintana Roo, México. Eggs were hatched in deoxygenated water from egg papers and then fed brewer's yeast. Adults were provided 10% (w/v) sucrose solution and were blood fed on citrated sheep blood in an artificial membrane feeder every three days. Incubators were set to a 14∶10 photoperiod, 30°C water temperature for larvae and 28°C for adult with a relative humidity of 85%.

### Bioassays and temephos selection

F_2_ or F_3_ offspring from the field constituted the F_S0_ generation in the selection experiments. F_S0_ larvae were bioassayed to estimate the concentration of temephos (Chem Service, West Chester, PA) necessary to kill 50% of larvae (LC_50_). Bioassays were performed in plastic cups containing 100 ml of water with five different concentrations of temephos in 1 mL ethanol as a solvent. Approximately 25 3^rd^-instar larvae were gently pipetted into each cup. Mortality was recorded every 15 minutes up to two hours. All larvae were then transferred into clean water and mortality was scored at 24 hours. Each bioassay was performed in triplicate to obtain ∼75 larvae per concentration. LC_50_ and confidence limits were calculated using the IRMA quick calculator software (http://sourceforge.net/projects/irmaproj/files/Qcal/beta/QCal_ver_0.1_rev190.msi/download) which performs logistic regression [30].

Selection proceeded in three replicate lines for three generations. In the first round of selection 40–100 third instar larvae from each of the three replicates were exposed to an LC_50_ of 30 ng temephos/mL for two hours. Larvae were then transferred to clean water and mortality was recorded at 24 hours. Surviving larvae were transferred to 1 cubic foot rearing cages (BugDorm-1, Mega View Science, Co.) and raised to adults who were then blood fed to obtain F_S1_ eggs. We performed an initial bioassay with ∼75 larvae in each of the subsequent F_S1_–F_S3_ generations of selection to calculate the new LC_50_. From 40–100 larvae from each replicate were then exposed to the new LC_50_.

### Mapping family crosses

For the P_1_ mapping family, we crossed Solidaridad (SLD) F_S3_ and Iquitos (Iq) adults. Twenty P_1_♀SLD F_S3_×♂Iq and twenty reciprocal P_1_♀Iq×♂ SLD F_S3_ crosses were made. Larvae from each line were hatched and at the pupal stage, a female (larger size) from one strain was transferred to plastic cups in cardboard containers with a male pupa from the other strain. After adults emerged, they were allowed to mate for 3 days and the P_1_ male was frozen and held at −80°C. Females were blood fed three times with an artificial membrane feeder over the next ten days and the P_1_ female was then frozen and held at −80°C. Egg batches were maintained at room temperature for 7 days and then hatched by submersion in water followed by feeding them on Brewer's yeast *ad libidum*. For the F_1_ intercross families, one female and one male pupa from the same P_1_ family were allowed to emerge, mate and blood fed to eventually generate F_2_ progeny. F_2_ eggs from the largest F_1_ families were hatched and siblings were intercrossed in a single cage.

### Resistance phenotyping of mapping families

Third instar larvae (200 total) were exposed to 250 ng temephos/mL. After 2 hours, larvae that were unresponsive to prodding with a pipette tip were individually transferred to a labeled 1.5 mL microcentrifuge tube and frozen at −80°C. This was repeated every two hours for the next 16 hours. After 16 hours all remaining larvae were recorded as resistant.

### DNA extraction

The DNA of the P_1_ and F_1_ parents, and the two sets of 200 F_3_ offspring was individually isolated following the salt extraction method [31] and then suspended in 200 µl of TE buffer (10 mM Tris-HCl, 1 mM EDTA pH 8.0). The DNA was divided into 2–100 µl aliquots and stored at −80°C.

### PCR of cDNA-Single Strand Conformation Polymorphisms (SSCP) markers

A total of 23 single copy genes [32], [33] and 26 microsatellite loci from [34] were amplified and analyzed. Each of these 49 genes has a known physical and linkage map position in the *Ae. aegypti* genome. A PCR mixture sufficient to perform 100 25-µl reactions was made by mixing 2,114 µL ddH_2_O, 250 µL 10×Taq buffer (500 mM KCl, 100 mM Tris-HCL pH 9.0), 25 µL of 20 mM dNTPs, and 2,500 pm of each of the primers. This reaction mixture was set under a UV light source (302 nm) for 10 min, after which 20 µl of Taq DNA polymerase was added. The mixture was then dispensed into a 96-well plate. Template DNA (∼100 ng) was then added to each well, followed by a drop of sterilized mineral oil. Each set of reactions was checked for contamination by the use of a negative control containing all reagents except template DNA. Samples were stored at 4°C before electrophoresis. The contents of each well were tested for the presence of amplified products by loading 5 µl from each well onto a 1.5% (w/v) agarose gel made with Tris-Borate-EDTA buffer. DNA fragments were size fractionated by electrophoresis for 15–20 min at 112 V. Fragments were visualized by staining with Syber Green and viewing the gel over a UV transilluminator. SSCP analysis and silver staining procedures were previously published [31].

### Melting curve assay for SNP

Polymorphic SSCP-markers were sequenced in the four P_1_ and F_1_ parents to test for SNPs and to determine the inheritance patterns of SNP alleles. Sequences were aligned using CLUSTALW [35]. Allele specific primers were designed at those loci in which genotypes were fully or partially informative in the P_1_ and F_1_ parents. Design of primers for melting curve PCR is previously published [36]. Allele specific fragments were detected by melting curve PCR in a CFX-96 Real time PCR detection system (Bio-Rad, Hercules, CA). Table S1 provides previously unpublished oligonucleotide sequences for allele specific detection.

### Quantitative trait loci (QTL) analyses

Associations between genotypes at each marker locus and hours until death (HTD) phenotype were initially assessed with ANOVA using summary (glm(HTD∼“Marker locus name”)) in R2.15.2 [37]. Our null hypothesis was that HTD was equal in each genotype. Associations between death (scored 0) or survival (1) (DOA) after 16 hours were initially assessed with Fisher's exact test (table (DOA, “Marker locus name”)) in R2.15.2. The null hypothesis was that the proportions of surviving larvae were equal in each genotype class. When the ANOVA or Fisher's exact test yielded a probability below 0.05, we examined the inheritance of the alleles at that locus. Our *a priori* hypothesis was that an excess of F_3_ individuals with an allele inherited from the SLD P_1_ parent would be resistant while an excess of F_3_ individuals with an allele inherited from the Iq P_1_ parent would die.

Multiple Interval mapping (MIM) [38] was then performed using QTL Cartographer 2.5 [39]. Two separate MIM were done. First, mosquitoes were scored as 2, 4, 6, 8, 10, 12, 14, 16 or 24 corresponding to hours until death. Second, F_3_ mosquitoes were scored as one if they survived to 16 hours or as zero if they died before 16 hours. In either case we created an initial model containing QTL map positions for markers at which ANOVA or Fisher's exact tests were significant. This model was then refined in MIM by 1) searching for new QTL, 2) estimating QTL effects, 3) obtaining and recording a summary, 4) optimizing QTL position, 5) searching for new QTL interactions, 6) testing for existing QTL main effects, 7) testing for existing QTL interaction effects, and 8) obtaining and recording a final summary. In addition, we used QTL Cartographer 2.5 to perform an initial MIM model selection on all markers using forward and backward selection with a significance level criterion of 0.01. We then compared this model with the model based upon markers identified as significant by ANOVA or Fisher's exact tests. The models agreed in all four cases: (1) ♀ SLD F_S3_×♂Iq –HTD (2) ♀ SLD F_S3_×♂Iq –DOA, (3) P_1_ ♀ Iq×♂ SLD – HTD and (4) P_1_ ♀ Iq×♂ SLD – DOA.

## Results

### Bioassays and selection

The concentration of temephos sufficient to kill 50% of larvae (LC_50_) was 50 ng temephos/mL water for the Iquitos strain. The Solidaridad F_S0_ strain initially had an LC_50_ of 27 ng temephos/mL water. Following three generations of temephos selection, the LC_50_ increased to 7.9 ug temephos/mL water in the Solidaridad strain. Thus the selected Solidaridad strain had ∼160 fold higher temephos resistance than the Iquitos strain. Among the SLD×Iq F_3_ larvae the LC_50_ was 6.5 ug temephos/mL water and was 1.9 ug temephos/mL water among the IqxSLD F_3_ larvae.

### Statistical analyses of phenotype × genotype associations

**Figure 1:**
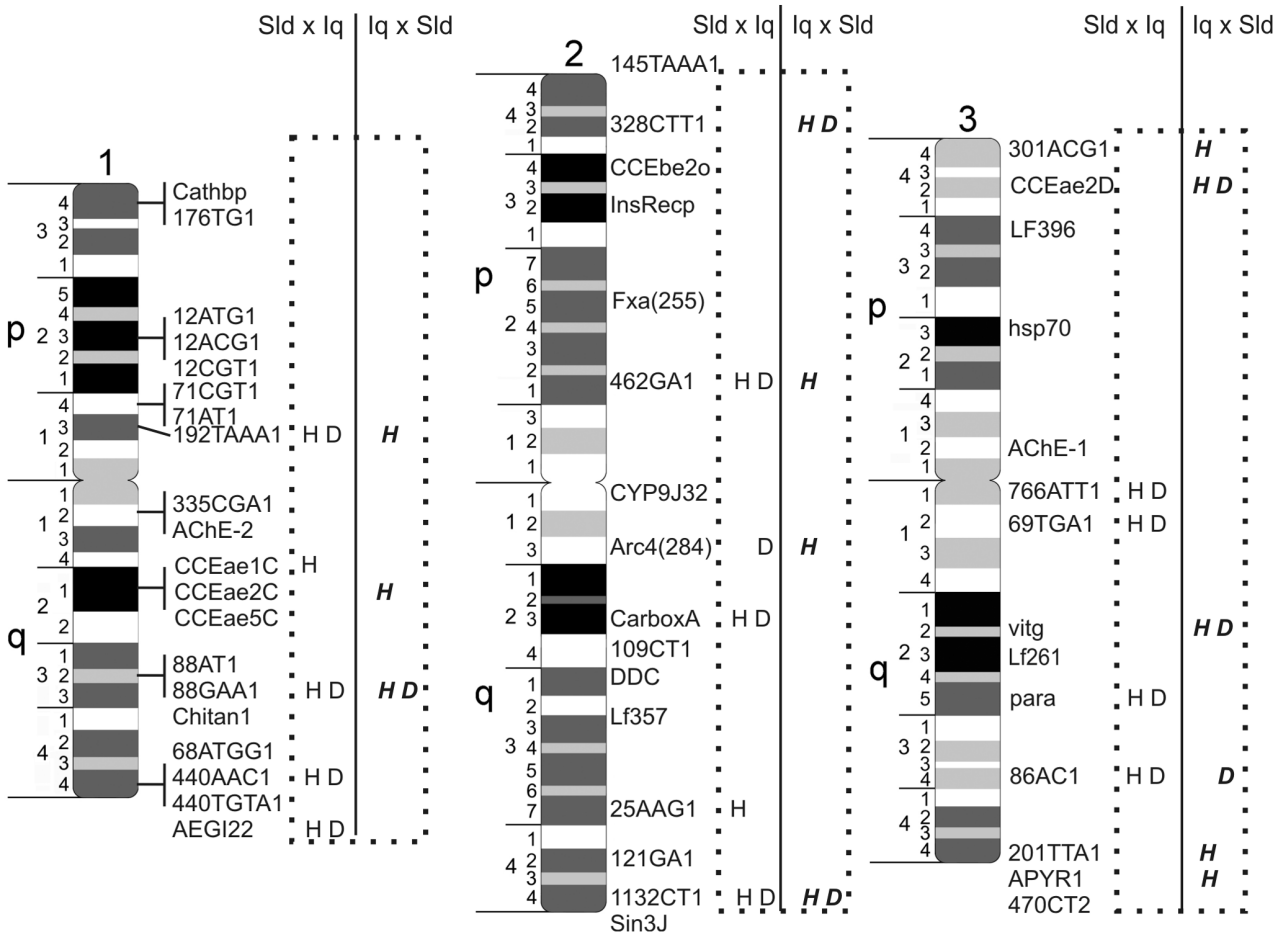
Physical positions of markers and QTL affecting hours until death (H) and survival (D). Physical markers correspond to the system described in [52].

The genetic markers used in constructing maps in both the SLDxIq and IqxSLD crosses are listed along with their linkage positions in Table S2. Results of the ANOVA to test the null hypothesis that time until death is equal among genotypes are presented in Table 1. Results of Fisher's Exact Test on proportions of surviving larvae among genotype classes appear in Table 2. Loci with significant results are shown for all three chromosomes in Figure 1.

**Table 1:** Analysis of Variance (ANOVA) of the hours until death among the three genotype classes.

Chromosome position (cM)	Marker Name	Hours until Death			ANOVA Prob.	Predicted correlation?
		Iq/Iq	SLD/Iq	SLD/SLD		
**SLD×Iq**						
Chromosome 1						
0.0	CathepB	13.6	13.5	13.7	0.9894	
0.3	176TG1	13.9	13.5	13.2	0.8889	
18.8	12ATG1	16.3	13.4	11.9	0.0543	
18.8	12CGT1	11.2	13.6	15.5	0.0610	
26.9	71CGT1	12.3	14.6	15.3	0.1162	
29.6	192TAAA1	10.2	13.2	19.3	0.0008	+
40.4	335CGA1	15.4	13.4	17.6	0.0523	
40.7	AChE-2	12.7	13.7	-	0.6185	
48.5	CCEae1C	9.6	12.9	15.0	0.0486	+
48.5	CCEae2C	9.7	13.3	14.5	0.2070	
48.5	CCEae5C	13.7	13.6	-	0.9047	
56.5	88AT1	-	13.0	13.7	0.6358	
56.5	88GAA1	12.9	13.3	19.5	0.0008	+
56.8	Chitan1	13.6	13.4	13.7	0.9674	
69.7	440AAC1	-	12.7	15.4	0.0108	+
70.0	AEGI22	19.5	12.4	-	0.0000	−
Chromosome 2						
0.0	45TAAA1	14.6	12.7	-	0.0717	
29.2	462GA1	9.3	16.7	24.0	0.0000	+
40.8	Arc4	-	13.8	12.8	0.5196	
46.7	Carbox	9.7	13.9	19.5	0.0000	+
48.6	109CT1	11.6	13.0	12.0	0.5610	
62.2	25AAG1	-	10.1	14.0	0.0299	+
66.1	121GA1	11.7	13.6	16.0	0.3073	
69.8	1132CT1	7.9	13.5	23.5	0.0000	+
70.0	Sin3J	12.6	13.9	14.6	0.3507	
Chromosome 3						
0.0	301ACG1	13.5	14.1	-	0.6036	
10.2	LF396	12.7	14.3	12.3	0.2491	
18.3	hsp70	-	13.9	14.3	0.9081	
30.4	AChE-1	12.7	13.7	-	0.6185	
32.5	766ATT1	12.2	15.8	-	0.0011	+
34.5	69TGA1	16.4	12.1	14.1	0.0021	−
44.7	LF261	14.5	13.5	12.7	0.5103	
48.8	para	15.2	12.1	14.6	0.0206	−
56.9	86AC1	9.3	14.6	8.9	0.0003	−
64.8	470CT2	14.1	14.0	12.6	0.4477	
**Iq×SLD**						
Chromosome 1						
0.0	CathepB	9.8	9.3	8.2	0.5813	
0.3	176TG1	8.1	8.6	9.7	0.4134	
18.8	12ACG1	10.1	8.1	-	0.0541	
18.8	12ATG1	-	9.3	8.1	0.3882	
18.8	12CGT1	8.4	9.9	8.4	0.3221	
26.9	71CGT1	8.6	9.3	10.7	0.6232	
26.9	71AT1	10.3	9.6	8.8	0.6318	
29.6	192TAAA1	5.5	9.6	9.7	0.0337	+
48.5	CCEae1C	8.8	9.1	9.3	0.9562	
48.5	CCEae2c	7.3	9.9	10.6	0.0230	+
48.5	CCEae5C	9.8	9.2	8.9	0.8247	
56.5	88GAA1	10.5	7.6	16.8	0.0000	−
56.5	88AT1	8.2	9.9	8.5	0.3426	
69.6	68ATGG1	8.5	9.7	4.8	0.2542	
69.7	440TGTA1	5.8	9.1	11.1	0.1577	
Chromosome 2						
5.8	328CTT1	-	7.2	11.6	0.0002	+
9.7	CCEbe20	8.9	9.1	-	0.8819	
13.6	insrecp	-	9.5	8.4	0.6120	
21.4	fxa	6.8	9.1	-	0.0739	
29.2	462GA1	-	7.5	4.9	0.0227	−
36.9	Cyp9J32	6.7	9.4	9.8	0.1143	
40.8	Arc4	-	9.7	6.3	0.0077	−
46.7	Carbox	9.0	8.7	11.2	0.2890	
48.6	109CT1	9.6	8.9	-	0.5126	
50.6	DDC	-	9.4	9.2	0.8670	
54.4	LF357	9.0	9.5	-	0.6926	
66.1	121GA1	9.6	8.9	7.0	0.1776	
69.8	1132CT1	4.9	9.7	24.0	0.0000	+
70.0	Sin3J	-	9.1	10.4	0.4016	
Chromosome 3						
0.0	301ACG1	7.8	8.2	24.0	0.0251	+
6.1	CCEae2D	12.5	8.6	-	0.0203	−
18.3	hsp70	-	9.5	6.7	0.3226	
30.4	AChE-1	-	8.6	9.8	0.5156	
34.5	69TGA1	9.7	9.7	8.8	0.9058	
42.7	vitg	16.0	8.7	-	0.0000	−
44.7	LF261	-	9.3	6.2	0.1521	
56.9	86AC1	7.6	10.2	9.3	0.1329	−
64.6	201TTA1	8.1	10.1	-	0.0361	−
64.8	470CT2	12.4	8.8	10.9	0.0950	
65.0	Apyr1	11.2	8.3	8.5	0.0216	−

The means among the three classes are listed as are the probabilities estimated in the ANOVA. Probabilities from the ANOVA are listed in the sixth column. The last column indicates whether the allele inherited from the SLD F_S3_ P_1_ parent conferred resistance while the allele inherited from the Iq P_1_ parent were susceptible.

**Table 2:** Fisher's Exact Test (FET) of proportions surviving past 16 hours among the three genotypes.

Chromosome position (cM)	Marker Name	Proportion surviving			Exact Test Prob.	Predicted correlation?
		Iq/Iq	SLD/Iq	SLD/SLD		
Chromosome 1						
**SLD×Iq**						
0.0	CathepB	0.333	0.281	0.282	1.0000	
0.3	176TG1	0.333	0.275	0.263	0.6539	
18.8	12ATG1	0.300	0.321	0.176	0.2774	
18.8	12CGT1	0.281	0.252	0.438	0.1194	
26.9	71CGT1	0.254	0.340	0.364	0.4337	
29.6	192TAAA1	0.091	0.268	0.632	0.0022	+
40.4	335CGA1	0.464	0.277	0.478	0.0677	
40.7	AChE-2	0.214	0.299	-	0.7611	
48.5	CCEae1C	0.000	0.277	0.346	0.0562	+
48.5	CCEae2C	0.000	0.291	0.316	0.3045	
48.5	CCEae5C	0.320	0.267	-	0.4388	
56.5	88AT1	-	0.222	0.301	0.4980	
56.5	88GAA1	0.244	0.256	0.714	0.0002	+
56.8	Chitan1	0.304	0.255	0.429	0.4800	
69.7	440AAC1	-	0.234	0.381	0.0379	+
70.0	AEGI22	0.714	0.211	-	0.0000	−
Chromosome 2						
0.0	145TAAA1	0.352	0.241	-	0.1165	
29.2	462GA1	0.000	0.525	1.000	0.0000	+
40.8	Arc4	-	0.327	0.080	0.0097	−
46.7	Carbox	0.101	0.267	0.650	0.0000	+
48.6	109CT1	0.188	0.295	0.143	0.0586	
62.2	25AAG1		0.158	0.304	0.2869	+
66.1	121GA1	0.286	0.274	0.526	0.0782	
69.8	1132CT1	0.150	0.207	0.964	0.0000	+
70.0	Sin3J	0.194	0.329	0.360	0.0988	
Chromosome 3						
0.0	301ACG1	0.275	0.337	-	0.4279	
10.2	LF396	0.255	0.327	0.194	0.2980	
18.3	hsp70	-	0.316	0.375	0.7110	
30.4	AChE-1	0.214	0.299	-	0.7611	
32.5	766ATT1	0.227	0.400	-	0.0142	+
34.5	69TGA1	0.492	0.163	0.407	0.0000	−
44.7	LF261	0.333	0.284	0.267	0.7858	
48.8	para	0.351	0.212	0.407	0.0412	−
56.9	86AC1	0.000	0.353	0.050	0.0002	−
64.8	470CT2	0.381	0.305	0.230	0.3770	
**Iq×SLD**						
Chromosome 1						
0.0	CathepB	0.091	0.140	0.114	0.8592	
0.3	176TG1	0.100	0.115	0.169	0.5624	
18.8	12ACG1	0.135	0.118	-	0.8206	
18.8	12ATG1	-	0.140	0.077	0.5379	
18.8	12CGT1	0.136	0.163	0.047	0.1533	
26.9	71CGT1	0.174	0.125	0.235	0.3515	
26.9	71AT1	0.000	0.117	0.138	0.7957	
29.6	192TAAA1	0.000	0.123	0.171	0.1246	+
48.5	CCEae1C	0.125	0.138	0.121	0.9253	
48.5	CCEae2c	0.067	0.172	0.130	0.1865	+
48.5	CCEae5C	0.097	0.146	0.113	0.7965	
56.5	88GAA1	0.231	0.012	0.600	0.0000	−
56.5	88AT1	0.143	0.144	0.096	0.7535	
69.6	68ATGG1	0.050	0.176	0.000	0.3281	
69.7	440TGTA1	0.000	0.115	0.278	0.1000	
Chromosome 2						
5.8	328CTT1	-	0.065	0.253	0.0034	+
9.7	CCEbe20	0.091	0.134	-	0.4755	
13.6	insrecp	-	0.133	0.100	1.0000	
21.4	fxa	0.032	0.138	-	0.1254	
29.2	462GA1	0.090	0.000	-	0.3349	−
36.9	Cyp9J32	0.038	0.146	0.134	0.4101	
40.8	Arc4	-	0.145	0.031	0.0863	−
46.7	Carbox	0.125	0.093	0.217	0.3054	
48.6	109CT1	0.161	0.112	-	0.3614	
50.6	DDC	-	0.118	0.144	0.6672	
54.4	LF357	0.160	0.134	­	0.7547	
66.1	121GA1	0.164	0.090	0.033	0.1415	
69.8	1132CT1	0.000	0.120	1.000	0.0000	+
70.0	Sin3J	-	0.131	0.100	1.0000	
Chromosome 3						
0.0	301ACG1	0.100	0.074	1.000	0.0788	+
6.1	CCEae2D	0.313	0.098	-	0.0253	−
18.3	hsp70	-	0.142	0.000	1.0000	
30.4	AChE-1	-	0.098	0.182	0.3177	
34.5	69TGA1	0.167	0.157	0.154	1.0000	
42.7	vitg	0.438	0.108	-	0.0019	−
44.7	LF261	-	0.132	0.000	0.6161	
56.9	86AC1	0.000	0.200	0.138	0.0394	−
64.6	201TTA1	0.143	0.110	-	0.5247	−
64.8	470CT2	0.250	0.119	0.000	0.2132	
65.0	Apyr1	0.193	0.098	0.103	0.2245	−

The means in each of the three genotypes are listed. Probabilities from the Exact Test are listed in the sixth column. The last column indicates whether the allele was inherited from the SLD F_S3_ P_1_ parent conferred resistance while the allele inherited from the Iq P_1_ parent was associated with susceptibility.

In the SLDxIq cross there were five QTL on chromosome 1 associated with HTD, four on chromosome 2 and four on chromosome 3. In the same cross there were four QTL on chromosome 1 associated with DOA, four on chromosome 2 and four on chromosome 3. In the IqxSLD cross there were three QTL on chromosome 1 associated with HTD, four on chromosome 2 and five on chromosome 3. There was one QTL on chromosome 1 associated with DOA, two on chromosome 2 and three on chromosome 3. The two families shared common QTL at loci 192TAAA1 and 88GAA1 on chromosome 1, at loci 462GA1 and 1132CT1 on chromosome 2 and at locus 86AC1 on chromosome 3. Between the two families there were six, six and nine QTL affecting HTD on chromosomes 1, 2, and 3, respectively or 21 loci in total. In the two families there were four, five and six QTL affecting DOA on chromosomes 1, 2, and 3, respectively or 15 loci in total.

**Figure 2:**
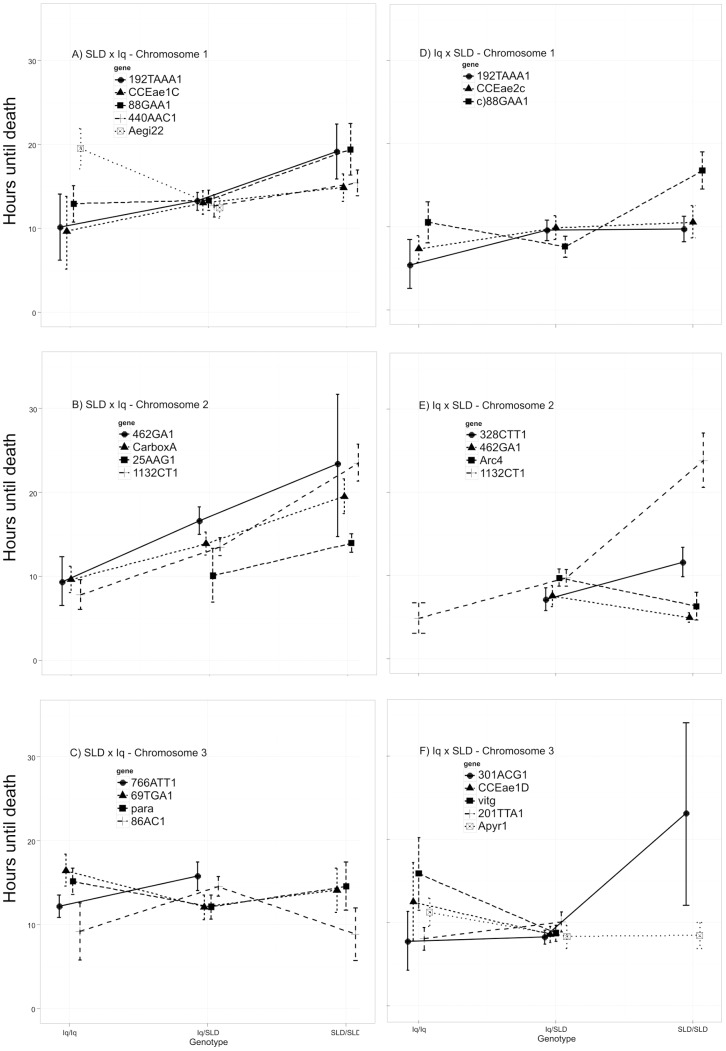
Hours until death among larvae plotted against the three possible genotypes at each of the markers found to be significantly associated with the HTD phenotype (Table 1). Iq/Iq = both alleles inherited from the Iquitos parent, Iq/SLD = heterozygous for alleles inherited from both Iquitos and Solidaridad parents, SLD/SLD = both alleles inherited from the Solidaridad parent. The second column corresponds to chromosomes 1, 2, and 3 in the Iq×SLD cross. Error bars are Bayes 95% highest density intervals (HDI), credible differences exist when the 95% HDI fail to overlap. For the SLD×Iq cross, A) shows the relationship among genotypes at six loci on chromosome 1 and HTD, B) is the relationship among genotypes at four loci on chromosome 2 and HTD, and C) indicates the relationship among genotypes at four loci on chromosome 3 and HTD. For the Iq×SLD cross, D) shows the relationship among genotypes at three loci on chromosome 1 and HTD, E) is the relationship among genotypes at four loci on chromosome 2 and HTD, and F) indicates the relationship among genotypes at five loci on chromosome 3 and HTD.

When the ANOVA or Fisher's exact tests yielded a probability below 0.05, we examined the inheritance of the alleles at that locus. The last columns of Tables 1 and 2 indicate when the allele inherited from the SLD F_S3_ P_1_ parent were associated with resistance while the allele inherited from the Iq P_1_ parent was associated with susceptibility. Figure 2 plots HTD among larvae with the three possible genotypes. The first column of plots correspond to chromosomes 1, 2, and 3 in the SLDxIq cross. SLD alleles conferred slightly greater longevity for the first three marker loci on chromosome 1 but Aegi22 Iq homozygotes had greater longevity than heterozygotes (Fig. 2A). In contrast, SLD alleles confer greater longevity for all marker loci on chromosome 2 (Fig. 2B) and the effects appear to be additive. On chromosome 3, no general trend is evident (Fig. 2C). Iq homozygotes confer slightly greater longevity at marker loci 69TGA1 and para. The opposite trend is seen in markers 766ATT1 and 86AC1.

The second column in Figure 2 corresponds to chromosomes 1, 2, and 3 in the Iq×SLD cross. Again, SLD alleles confer slightly greater longevity on chromosome 1 (Fig. 2D). In contrast, on chromosome 2 SLD alleles at markers 328CTT1, 462GA1, and Arc4 confer only slightly greater longevity (Fig. 2E) while SLD alleles at the 1132CT1 locus appear to act as recessives in conferring much greater longevity. A similar pattern is seen in SLD alleles at 301ACG1 on chromosome 3 (Fig. 2F). However, Iq homozygotes confer slightly greater longevity at marker loci CCEae2D, vitg, 201TTA1 and Apyr1.

**Figure 3:**
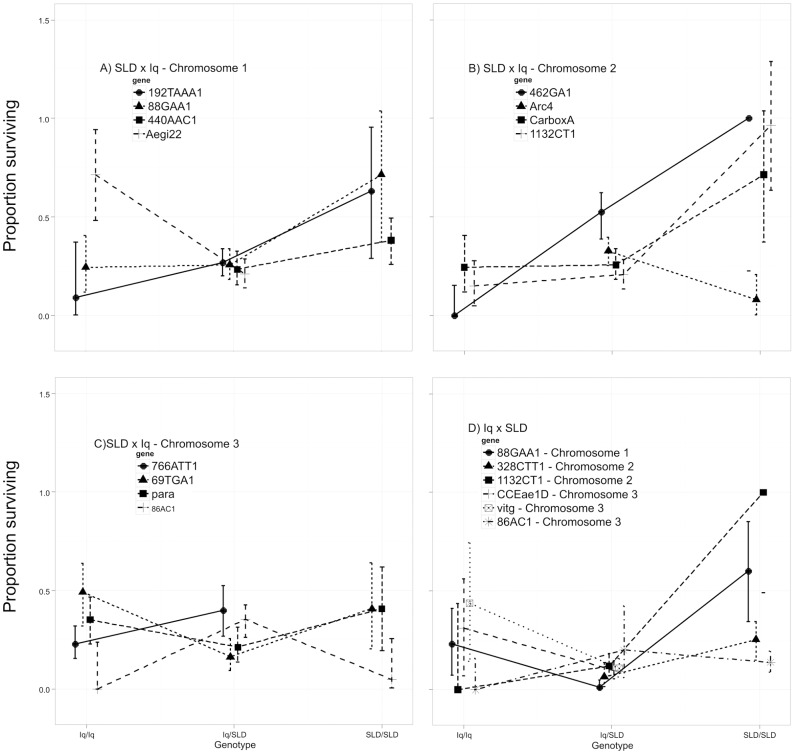
Proportion surviving among larvae plotted against the three possible genotypes at each of the markers found to be significantly associated with the DOA phenotype (Table 2). Iq/Iq = both alleles inherited from the Iquitos parent, Iq/SLD = heterozygous for alleles inherited from both Iquitos and Solidaridad parents, SLD/SLD = both alleles inherited from the Solidaridad parent. Error bars are Bayes 95% highest density intervals (HDI), credible differences exist when the 95% HDI fail to overlap. For the SLD×Iq cross, A) shows the relationship among genotypes at four loci on chromosome 1 and proportion surviving, B) is the relationship among genotypes at four loci on chromosome 2 and proportion surviving, and C) indicates the relationship among genotypes at four loci on chromosome 3 and proportion surviving. For the Iq×SLD cross, D) shows the relationship among genotypes at one locus on chromosome 1, two loci on chromosome 2 and 3 loci on chromosome 3 and proportion surviving.

Figure 3 plots proportion surviving past 16 hours among larvae with the three possible genotypes. In the SLDxIq cross SLD alleles conferred greater survival at the first three marker loci on chromosome 1 but Aegi22 Iq homozygotes had greater longevity than heterozygotes (Fig. 3A). Note that these are the same markers as in Figure 2A, but with markers 192TAAA1, and 88GAA1. SLD alleles confer a 50% increase in survival. On chromosome 2 (Fig. 3B), with the exception of Arc4, SLD alleles at markers, 462GA1, Carbox and 1132CT1 all greatly increase survival. SLD alleles at 462GA1 appear to act additively in increasing survival from zero in Iq homozygotes to 50% in heterozygotes to 100% in SLD homozygotes. Resistant alleles at markers Carbox and 1132CT1 are recessive with 75–80% greater survival in SLD homozygotes. As with HTD, on chromosome 3 there is no general trend (Fig. 3C). Iq homozygotes confer slightly greater survival at marker loci 69TGA1 and para but the opposite trend is seen in markers 766ATT1 and 86AC1. In the Iq×SLD cross (Fig. 3D) SLD alleles at marker 88GAA1 increase survival by 50% and SLD alleles appear recessive. Similarly, alleles at the 1132CT1 marker increased survival by 90%. Identical patterns were seen in the SLDxIq cross (Fig. 3B). On chromosome 3, Iq homozygotes confer slightly greater survival at marker loci CCEae2D, vitg, and 86AC1.

### QTL analysis

The results of Multiple Interval Mapping with the HTD and DOA phenotypes are shown for both crosses in Table 3. Eleven QTL were identified in the SLD×Iq cross and these accounted for 68% of the phenotypic variance in HTD. There were nine QTL that accounted for 63% of the phenotypic variance in DOA. These nine were also all associated with HTD. The QTL that accounted for most (48%) of the genetic variation in HTD were at 47 cM and 70 cM on chromosome 2. The QTL that accounted for the most variation in DOA was at 62 cM on chromosome 2. QTL at 30 cM and 70 cM on chromosome 1 affected both phenotypes.

**Table 3:** Multiple-interval mapping estimates of QTL position and associated genetic, environmental, and phenotypic variances.

SLD x Iq	Hours until Death						Survival		
σ^2^_Genetic_	36.91	68.3%					0.130	63.0%	
σ^2^_Residual_	17.14	31.7%					0.076	37.0%	
σ^2^_phenotypic_	54.05						0.206		

		Marker	Position	LOD	Effect	Effect(%)	LOD	Effect	Effect(%)
Chromosome 1	Add.	192TAAA1	29.7	2.1	3.02	4.2	1.4	0.16	3.4
	Dom.			0.3	-1.30	0.8	0.3	-0.07	0.8
	Add.	88GAA1	56.6	0.1	0.52	0.4	0.6	0.10	1.5
	Dom.			0.6	-2.12	1.6	1.6	-0.22	4.4
	Add.	440AAC1	69.8	0.8	2.56	2.2	-	-	-
	Dom.			0.0	0.43	-0.2	-	-	-
	Add.	AEGI22	69.9	2.0	-3.49	6.5	3.1	-0.21	8.1
	Dom.			0.8	-2.37	2.9	1.6	-0.19	4.5
						18.4			22.7
Chromosome 2	Add.	462GA1	29.3	0.6	1.21	1.9	0.4	0.07	1.4
	Dom.			1.9	3.25	4.8	1.4	0.17	5.0
	Add.	Carbox	46.8	8.4	3.69	17.4	0.0	-0.02	-0.2
	Dom.			0.3	0.86	0.2	0.3	0.14	-0.7
	Add.	25AAG1	62.3	0.1	5.00	3.4	5.5	0.25	17.1
	Dom.			0.1	-2.69	-1.5	0.2	-0.05	0.3
	Add.	1132CT1	69.9	7.0	4.21	15.5	4.1	0.20	10.1
	Dom.			0.6	1.54	0.7	0.0	0.00	0.0
						42.4			33.0
Chromosome 3	Add.	69TGA1	34.6	0.1	-0.34	0.4	0.1	-0.02	0.3
	Dom.			0.3	-0.90	1.2	1.2	-0.12	3.7
	Add.	para	48.9	0.7	-1.11	0.9	-	-	-
	Dom.			0.5	-1.28	1.7	-	-	-
	Add.	86AC1	57.0	0.3	-0.95	0.5	0.1	-0.02	0.1
	Dom.			0.9	1.93	2.8	1.1	0.14	3.2
						7.5			7.3
						68.3			63.0
**Iq x SLD**									
σ^2^_Genetic_	25.06	57.6%					0.034	31.3%	
σ^2^_Residual_	18.43	42.4%					0.075	68.7%	
σ^2^_phenotypic_	43.49						0.109		

		Marker	Position	LOD	Effect	Effect(%)	LOD	Effect	Effect(%)
Chromosome 1	Add.	192TAAA1	29.7	0.4	-0.84	1.0	-	-	-
	Dom.			0.5	1.24	0.6	-	-	-
	Add.	CCEae2c	48.6	0.6	-1.38	2.2	-	-	-
	Dom.			0.0	0.47	0.0	-	-	-
	Add.	88GAA1	56.6	0.9	-1.27	2.8	3.55	-0.15	8.7
	Dom.			1.7	-3.13	6.4	8.1	-0.30	20.4
						13.0			29.1
Chromosome 2	Add.	328CTT1	0.1	2.1	-1.95	5.6	-	-	-
	Dom.			0.7	-1.65	3.2	-	-	-
	Add.	462GA1	23.5	0.1	-0.48	0.0	-	-	-
	Dom.			0.5	2.04	2.1	-	-	-
	Add.	Arc4	35.1	0.0	0.02	0.0	-	-	-
	Dom.			0.1	1.67	1.1	-	-	-
	Add.	1132CT1	64.1	1.9	-4.04	11.2	-	-	-
	Dom.			0.0	-0.47	0.3	-	-	-
						23.5			
Chromosome 3	Add.	301ACG1	0.1	0.7	-3.65	1.4	-	-	-
	Dom.			0.2	-2.01	1.3	-	-	-
	Add.	CCEae2D	6.2	0.1	-2.53	-0.8	-	-	-
	Dom.			0.1	-5.34	5.2	-	-	-
	Add.	vitg	42.8	1.7	4.79	9.2	0.01	0.03	0.4
	Dom.			0.2	-1.69	2.5	0.06	0.11	1.8
	Add.	201TTA1	64.9	0.1	0.55	0.8	-	-	-
	Dom.			0.2	-1.54	1.8	-	-	-
						21.4			2.2
						57.9			31.3

Additive and dominance effects associated with hours until death and survival QTL in *Aedes aegypti* are also listed.

Genetic factors accounted for less of the variation in HTD and DOA phenotypes in the Iq×SLD cross. Eleven QTL were identified that accounted for 58% of the phenotypic variance in HTD. There were only two QTL that accounted for 31% of the variance in DOA and these were also associated with HTD. The QTL that accounted for most of the variation in HTD were at 57 cM on chromosome 1, 64 cM on chromosome 2 and 43 cM on chromosome 3. The only QTL that accounted for negligible variation in DOA was at 62 cM on chromosome 2. QTL at 57 cM on chromosome 1 affected both phenotypes. QTL at 30 and 57 cM on chromosome 1, and at 23.5 and 70 cM on chromosome 2 were common to both families

## Discussion

QTL mapping indicates that resistance to temephos is conditioned by many regions of the *Ae. aegypti* genome and therefore appears to behave as a classic quantitative genetic trait that is controlled by many loci each of minor effect. This pattern is supported by a recent parallel study in which we tracked changes in transcription of metabolic detoxification genes using the *Ae. aegypti* ‘Detox Chip’ microarray [40] during five generations of temephos selection [41]. We selected for temephos resistance in three replicates in each of six collections, five from México, and one from Peru. We used the esterase inhibitor DEF (S.S.S-tributylphosphorotrithioate) to show that esterases were the major metabolic source of resistance. However, the microarray data indicated that expression of many esterase genes increased with selection and that no single esterase was consistently upregulated among the six selected lines.

Target site resistance in acetylcholine esterase genes is a very common mechanism of resistance to organophosphate and carbamate insecticides [42]. We therefore tested for a significant genotype -phenotype interaction with SNPs in the AChE-2 gene (AAEL012141) at 40.7 cm on chromosome 1 and the AChE-1 gene (EF209048) at 3p1.2 (30.4 cM) on chromosome 3 [43]. Results in Table 1–3 show that no significant associations were detected. Similar studies of temephos resistance in field populations of *Ae. aegypti* also failed to detect insensitive acetylcholine esterase [44] despite the fact that these authors were able to generate recombinant clones that produced *Ae. aegypti* insensitive acetylcholine esterases in the laboratory [45]. Another possibility is that temephos in particular fails to select for insensitive acetylcholine esterases. Cuban investigators were able to select *Ae. aegypti* with 13-fold increase in insensitive acetylcholine esterase but using the carbamate insecticide propoxur [46].

Previous studies of esterase isozyme loci identified two genetically mapped loci associated with resistance to the organophosphate insecticide malathion. Elevated activity staining of Esterase-5 located at 57 cM at the base of Chromosome 1 [47] was reported [48]. This may correspond to the 57 cM QTL on chromosome 1 associated with marker 88GAA1 in both families in the current study. Similarly elevated activity staining of Esterase-6 located at 83 cM at the base of Chromosome 2 in the map of [47] was reported [49], [50]. This may correspond to the QTL at 70 cM on chromosome 2 associated with marker 1132CT1 found in both families in the current study. We have no means to formally check these associations because neither the nucleotide nor amino acid sequences of proteins Esterase-5 and 6 are known.

There are 49 currently identified carboxy/choline esterase genes [40]. With the recent publication of a physical map that contains 45% of the Ae. Aegypti genome [51], [52] we had hoped to learn the physical locations of many of these esterases. However, other than AChE-1 and AChE-2, there were only six other esterase genes that occurred in mapped supercontigs. These were CCEbe2o (AAEL008757) on 2p3.4 (also mapped in the present study see Figure 1), CCEjhe2o (AAEL004323) on 2q2.4, and four (CCEjhe1F (AAEL005200), CCEjhe2F (AAEL005198), CCEjhe3F (AAEL005210), and CCEjhe4F (AAEL005182)) all located in supercontig 1.145 at 2p4.4. Whether these four are associated with the QTL at 5.8 cM on the top of Chromosome 2 in the Qi×SLD cross (see Tables 1–2) is unknown at this time.

Even though the selected Solidaridad strain had overall ∼160 fold higher temephos resistance than the Iquitos strain, this pattern wasn't uniform across the entire genome. This could have affected the locations and relative contributions of QTL. There are many instances in Tables 1 and 2 wherein the mosquitoes homozygous for markers from the “susceptible” Iquitos strain were more resistant than heterozygotes or homozygous for markers from the “resistant” SLD strain (note especially the bottom of chromosome 3 for both HTD and DOA). This counterintuitive outcome is probably a result of using Iquitos mosquitoes taken directly from the field without selecting for a more susceptible phenotype. However, it could also be associated with negative fitness effects associated with resistance alleles in the SLD strain that became concentrated during selection.

In our previous QTL mapping study [36] we found resistance to permethrin to be principally (91.8% of genetic effect in MIM) under the control of target site insensitivity in the voltage gated sodium channel gene (orthologue of *paralysis* in *Drosophila* [53]). We have shown that the genetic architecture underlying temephos resistance to be completely different with both families having up to 11 QTL affecting the HTD phenotype in both families and from 2–9 QTL affecting DOA. The practical implications of these findings are that selection for temephos resistance in the field is likely to involve many (principally esterase) loci. It is unlikely that the same genes will be involved in all field populations and that genetic drift may play a large part in determining which combinations of the 49 currently identified carboxy/choline esterase genes [40] become upregulated and assume responsibility for metabolic detoxification of temephos.

## Supporting Information

Table S1 Single nucleotide polymorphic markers, vector base ID (or gene bank accession number), SNP position from cDNA and oligonucleotide sequence. The nucleotide at the 3′ end of primers tagged with [5′-Long tail] and [5′-Short tail] correspond to the SNP of interest. [5′-Long tail] corresponds to the sequence 5′-GCGGGCAGGGCGGCGGGGGCGGGGCC-′3 and [5′-Short tail] to the sequence 5′-GCGGGC-3′. These GC rich tails produce amplicons that can be differentiated by melting curve PCR or agarose electrophoresis.
(DOCX)

Table S2 Names and locations of markers used in mapping of temephos resistance QTL in *Aedes aegypti*.
(DOCX)

## References

[1] Gubler DJ (2012) The Economic Burden of Dengue. American Journal of Tropical Medicine and Hygiene 86: 743–744. 22556068 10.4269/ajtmh.2012.12-0157 PMC3335674

[2] Sabchareon A, Wallace D, Sirivichayakul C, Limkittikul K, Chanthavanich P, et al (2012) Protective efficacy of the recombinant, live-attenuated, CYD tetravalent dengue vaccine in Thai schoolchildren: a randomised, controlled phase 2b trial. The Lancet 380: 1559–1567. 10.1016/S0140-6736(12)61428-7 22975340

[3] Halstead SB (2012) Dengue vaccine development: a 75% solution? Lancet 380: 1535–1536. 22975339 10.1016/S0140-6736(12)61510-4

[4] McAllister JC, Godsey MS, Scott ML (2012) Pyrethroid resistance in Aedes aegypti and Aedes albopictus from Port-au-Prince, Haiti. Journal of Vector Ecology 37: 325–332. 23181855 10.1111/j.1948-7134.2012.00234.x PMC4562397

[5] Somwang P, Yanola J, Suwan W, Walton C, Lumjuan N, et al (2011) Enzymes-based resistant mechanism in pyrethroid resistant and susceptible Aedes aegypti strains from northern Thailand. Parasitology Research 109: 531–537. 21336645 10.1007/s00436-011-2280-0

[6] Polson KA, Rawlins SC, Brogdon WG, Chadee DD (2011) Characterisation of DDT and Pyrethroid Resistance in Trinidad and Tobago populations of Aedes aegypti. Bulletin of Entomological Research 101: 435–441. 21272394 10.1017/S0007485310000702

[7] Kawada H, Higa Y, Komagata O, Kasai S, Tomita T, et al (2009) Widespread Distribution of a Newly Found Point Mutation in Voltage-Gated Sodium Channel in Pyrethroid-Resistant Aedes aegypti Populations in Vietnam. Plos Neglected Tropical Diseases 3. 10.1371/journal.pntd.0000527 PMC2754656 19806205

[8] Saavedra-Rodriguez K, Urdaneta-Marquez L, Rajatileka S, Moulton M, Flores AE, et al (2007) A mutation in the voltage-gated sodium channel gene associated with pyrethroid resistance in Latin American Aedes aegypti. Insect Molecular Biology 16: 785–798. 18093007 10.1111/j.1365-2583.2007.00774.x

[9] Garcia GP, Flores AE, Fernandez-Salas I, Saavedra-Rodriguez K, Reyes-Solis G, et al (2009) Recent Rapid Rise of a Permethrin Knock Down Resistance Allele in Aedes aegypti in Mexico. Plos Neglected Tropical Diseases 3: e527. 19829709 10.1371/journal.pntd.0000531 PMC2759509

[10] Henrick CA (2007) Methoprene. Journal of the American Mosquito Control Association 23: 225–239. 17853608 10.2987/8756-971X(2007)23[225:M]2.0.CO;2

[11] Mazzarri MB, Georghiou GP (1995) Characterization of Resistance to Organophosphate, Carbamate, and Pyrethroid Insecticides in-Field Populations of Aedes-Aegypti from Venezuela. Journal of the American Mosquito Control Association 11: 315–322. 8551300

[12] Rawlins SC, Wan JOH (1995) Resistance in Some Caribbean Populations of Aedes-Aegypti to Several Insecticides. Journal of the American Mosquito Control Association 11: 59–65. 7542312

[13] Wirth MC, Georghiou GP (1999) Selection and characterization of temephos resistance in a population of Aedes aegypti from Tortola, British Virgin Islands. Journal of the American Mosquito Control Association 15: 315–320. 10480122

[14] Rodriguez MM, Bisset J, De Fernandez DM, Lauzan L, Soca A (2001) Detection of insecticide resistance in Aedes aegypti (Diptera: Culicidae) from Cuba and Venezuela. Journal of Medical Entomology 38: 623–628. 11580033 10.1603/0022-2585-38.5.623

[15] Rodriguez MM, Bisset J, Ruiz M, Soca A (2002) Cross-resistance to pyrethroid and organophosphorus insecticides induced by selection with temephos in Aedes aegypti (Diptera: Culicidae) from Cuba. Journal of Medical Entomology 39: 882–888. 12495187 10.1603/0022-2585-39.6.882

[16] Jirakanjanakit N, Saengtharatip S, Rongnoparut P, Duchon S, Bellec C, et al (2007) Trend of temephos resistance in Aedes (Stegomyia) mosquitoes in Thailand during 2003–2005. Environmental entomology 36: 506–511. 17540057 10.1603/0046-225x(2007)36[506:totria]2.0.co;2

[17] Macoris MD, Andrighetti MTM, Takaku L, Glasser CM, Garbeloto VC, et al (2003) Resistance of Aedes aegypti from the State of Sao Paulo, Brazil, to organophosphates insecticides. Memorias Do Instituto Oswaldo Cruz 98: 703–708. 12973541 10.1590/s0074-02762003000500020

[18] Lima JBP, Da-Cunha MP, Da Silva RC, Galardo AKR, Soares SD, et al (2003) Resistance of Aedes aegypti to organophosphates in several municipalities in the state of Rio de Janeiro and Espirito Santo, Brazil. American Journal of Tropical Medicine and Hygiene 68: 329–333. 12685640

[19] Braga IA, Lima JBP, Soares SD, Valle D (2004) Aedes aegypti resistance to Temephos during 2001 in several municipalities in the states of Rio de Janeiro, Sergipe, and Alagoas, Brazil. Memorias Do Instituto Oswaldo Cruz 99: 199–203. 15250476 10.1590/s0074-02762004000200015

[20] Lima EP, de Oliveira AM, Lima JWD, Junior ANR, Cavalcanti LPD, et al (2006) Aedes aegypti resistance to temefos in counties of Ceara State. Revista Da Sociedade Brasileira De Medicina Tropical 39: 259–263. 16906249 10.1590/s0037-86822006000300006

[21] Beserra EB, Fernandes CRM, De Queiroga MDC, De Castro FP (2007) Resistance of Aedes aegypti (L.) (Diptera: Culicidae) populations to organophosphates ternephos in the Paraiba State, Brazil. Neotropical Entomology 36: 303–307. 17607466 10.1590/s1519-566x2007000200019

[22] Lazcano JAB, Rodriguez MM, Martin JLS, Romero JE, Montoya R (2009) Assessing the insecticide resistance of an Aedes aegypti strain in El Salvador. Revista Panamericana De Salud Publica-Pan American Journal of Public Health 26: 229–234. 20058833

[23] Marcombe S, Poupardin R, Darriet F, Reynaud S, Bonnet J, et al (2009) Exploring the molecular basis of insecticide resistance in the dengue vector Aedes aegypti: a case study in Martinique Island (French West Indies). Bmc Genomics 10: 494. 19857255 10.1186/1471-2164-10-494 PMC2770535

[24] Llinas GA, Seccacini E, Gardenal CN, Licastro S (2010) Current resistance status to temephos in Aedes aegypti from different regions of Argentina. Memorias do Instituto Oswaldo Cruz 105: 113–116. 20209341 10.1590/s0074-02762010000100019

[25] Seccacini E, Lucia A, Zerba E, Licastro S, Masuh H (2008) Aedes aegypti resistance to temephos in Argentina. Journal of the American Mosquito Control Association 24: 608–609. 19181076 10.2987/5738.1

[26] Tikar SN, Kumar A, Prasad GBKS, Prakash S (2009) Temephos-induced resistance in Aedes aegypti and its cross-resistance studies to certain insecticides from India. Parasitology Research 105: 57–63. 19229558 10.1007/s00436-009-1362-8

[27] Ocampo CB, Salazar-Terreros MJ, Mina NJ, McAllister J, Brogdon W (2011) Insecticide resistance status of Aedes aegypti in 10 localities in Colombia. Acta Tropica 118: 37–44. 21300017 10.1016/j.actatropica.2011.01.007

[28] Polson KA, Brogdon WG, Rawlins SC, Chadee DD (2011) Characterization of insecticide resistance in Trinidadian strains of Aedes aegypti mosquitoes. Acta Tropica 117: 31–38. 20858454 10.1016/j.actatropica.2010.09.005

[29] Polson KA, Rawlins SC, Brogdon WG, Chadee DD (2010) Organophosphate resistance in Trinidad and Tobago strains of Aedes aegypti. Journal of the American Mosquito Control Association 26: 403–410. 21290936 10.2987/10-6019.1

[30] Lozano-Fuentes S, Saavedra-Rodriguez K, Black WC, Eisen L (2012) Qcal: A Software Application for the Calculation of Dose-Response Curves in Insecticide Resistance Bioassays. Journal of the American Mosquito Control Association 28: 59–61. 22533088 10.2987/11-6192.1

[31] Black WC, DuTeau NM (1997) RAPD-PCR and SSCP analysis for insect population genetic studies. In: J C, CB B, C L, editors. The Molecular Biology of Insect Disease Vectors: A Methods Manual. New York: Chapman and Hall. pp. 361–373.

[32] Gomez-Machorro C, Bennett KE, Munoz MD, Black WC (2004) Quantitative trait loci affecting dengue midgut infection barriers in an advanced intercross line of Aedes aegypti. Insect Molecular Biology 13: 637–648. 15606812 10.1111/j.0962-1075.2004.00522.x

[33] Fulton RE, Salasek ML, DuTeau NM, Black WC (2001) SSCP analysis of cDNA markers provides a dense linkage map of the Aedes aegypti genome. Genetics 158: 715–726. 11404335 10.1093/genetics/158.2.715 PMC1461678

[34] Lovin DD, Washington KO, deBruyn B, Hemme RR, Mori A, et al (2009) Genome-based polymorphic microsatellite development and validation in the mosquito Aedes aegypti and application to population genetics in Haiti. Bmc Genomics 10: 590. 20003193 10.1186/1471-2164-10-590 PMC3087561

[35] Thompson JD, Higgins DG, Gibson TJ (1994) Clustal-W - Improving the Sensitivity of Progressive Multiple Sequence Alignment through Sequence Weighting, Position-Specific Gap Penalties and Weight Matrix Choice. Nucleic Acids Research 22: 4673–4680. 7984417 10.1093/nar/22.22.4673 PMC308517

[36] Saavedra-Rodriguez K, Strode C, Suarez AF, Salas IF, Ranson H, et al (2008) Quantitative Trait Loci Mapping of Genome Regions Controlling Permethrin Resistance in the Mosquito Aedes aegypti. Genetics 180: 1137–1152. 18723882 10.1534/genetics.108.087924 PMC2567363

[37] Pinheiro J, Bates D, DebRoy S, Sarkar D (2013) nlme: Linear and Nonlinear Mixed Effects Models. The R Development Core Team. 3.1–108. ed.

[38] Zeng ZB (1994) Precision Mapping of Quantitative Trait Loci. Genetics 136: 1457–1468. 8013918 10.1093/genetics/136.4.1457 PMC1205924

[39] Wang S, Basten CJ, Zeng Z-B (2007) Windows QTL Cartographer 2.5. In: Department of Statistics NCSU, editor. Raleigh, NC.

[40] Strode C, Wondji CS, David JP, Hawkes NJ, Lumjuan N, et al (2008) Genomic analysis of detoxification genes in the mosquito Aedes aegypti. Insect Biochemistry and Molecular Biology 38: 113–123. 18070670 10.1016/j.ibmb.2007.09.007

[41] Saavedra-Rodriguez K, Strode C, Flores AE, Garcia-Luna S, Reyes-Solis G, et al (2013) Differential transcription profiles in Aedes aegypti detoxification genes after temephos selection. Insect Molecular Biology 23: 199–215. 24299217 10.1111/imb.12073 PMC4091897

[42] Vontas JG, Hejazi MJ, Hawkes NJ, Cosmidis N, Loukas M, et al (2002) Resistance-associated point mutations of organophosphate insensitive acetylcholinesterase, in the olive fruit fly Bactrocera oleae. Insect Molecular Biology 11: 329–336. 12144698 10.1046/j.1365-2583.2002.00343.x

[43] Mori A, Lobo NF, deBruyn B, Severson DW (2007) Molecular cloning and characterization of the complete acetylcholine sterase gene (Ace1) from the mosquito Aedes aegypti with implications for comparative genome analysis. Insect Biochemistry and Molecular Biology 37: 667–674. 17550823 10.1016/j.ibmb.2007.03.014 PMC2716755

[44] Vaughan A, Chadee DD, Ffrench-Constant R (1998) Biochemical monitoring of organophosphorus and carbamate insecticide resistance in Aedes aegypti mosquitoes from Trinidad. Medical and Veterinary Entomology 12: 318–321. 9737606 10.1046/j.1365-2915.1998.00111.x

[45] Vaughan A, Rocheleau T, ffrench-Constant R (1997) Site-directed mutagenesis of an acetylcholinesterase gene from the yellow fever mosquito Aedes aegypti confers insecticide insensitivity. Experimental Parasitology 87: 237–244. 9371089 10.1006/expr.1997.4244

[46] Bisset J, Rodriguez MM, Fernandez D (2006) Selection of insensitive acetylcholinesterase as a resistance mechanism in Aedes aegypti (Diptera: Culicidae) from Santiago de Cuba. Journal of Medical Entomology 43: 1185–1189. 17162951

[47] Munstermann LE, Craig GB (1979) Genetics of Aedes-Aegypti - Updating the Linkage Map. Journal of Heredity 70: 291–296.

[48] Field WN, Hitchen JM (1987) Linkage relationships between a low-mobility esterase locus and group I markers in larvae of the yellow fever mosquito, Aedes aegypti (Diptera: Culicidae). Journal of Medical Entomology 24: 512–514. 3625727 10.1093/jmedent/24.4.512

[49] Field WN, Hitchen JM (1981) Linkage relationships between an esterase locus and group II markers in the yellow fever mosquito, Aedes aegypti (Diptera: Culicidae). Journal of Medical Entomology 18: 61–64. 6945437 10.1093/jmedent/18.1.61

[50] Field WN, Hitchen JM, Rees AT (1984) Esterase activity in strains of Aedes aegypti (Diptera: Culicidae) tolerant and susceptible to the organophosphate insecticide malathion. Journal of Medical Entomology 21: 412–418. 6492084 10.1093/jmedent/21.4.412

[51] Timoshevskiy VA, Kinney NA, deBruyn BS, Mao CH, Tu ZJ, et al (2014) Genomic composition and evolution of Aedes aegypti chromosomes revealed by the analysis of physically mapped supercontigs. Bmc Biology 12: 27. 24731704 10.1186/1741-7007-12-27 PMC4021624

[52] Timoshevskiy VA, Severson DW, deBruyn BS, Black WC, Sharakhov IV, et al (2013) An Integrated Linkage, Chromosome, and Genome Map for the Yellow Fever Mosquito Aedes aegypti. Plos Neglected Tropical Diseases 7: e2052. 23459230 10.1371/journal.pntd.0002052 PMC3573077

[53] Suzuki DT, Grigliat T, Williams R (1971) Temperature-Sensitive Mutations in Drosophila-Melanogaster.7. Mutation (Parats) Causing Reversible Adult Paralysis. Proceedings of the National Academy of Sciences of the United States of America 68: 890–893. 5280526 10.1073/pnas.68.5.890 PMC389073

