# Supplementary material for: QTL Mapping of Genome Regions Controlling Temephos Resistance in Larvae of the Mosquito Aedes aegypti
Source: PLoS Negl Trop Dis. 2014 Oct 16;8(10):e3177. doi: 10.1371/journal.pntd.0003177 (PMC4199591; doi:10.1371/journal.pntd.0003177)
Supplement: Table S1 — Single nucleotide polymorphic markers, vector base ID (or gene bank accession number), SNP position from cDNA and oligonucleotide sequence. The nucleotide at the 3′ end of primers tagged with [5′-Long tail] and [5′-Short tail] correspond to the SNP of interest. [5′-Long tail] corresponds to the sequence 5′-GCGGGCAGGGCGGCGGGGGCGGGGCC-′3 and [5′-Short tail] to the sequence 5′-GCGGGC-3′. These GC rich tails produce amplicons that can be differentiated by melting curve PCR or agarose electrophoresis. (DOCX) [file pntd.0003177.s001.docx]

| Table S1- Single Nucleotide polymorphic markers, vector base ID (or gene bank accession number), SNP position from cDNA and oligonucleotide sequence. The nucleotide at the -‘3 end of primers tagged with [5'-Long tail ] and [5'-Short tail ] correspond to the SNP of our interest. [5'-Long tail ] corresponds to the sequence 5’-GCGGGCAGGGCGGCGGGGGCGGGGCC-‘3 and [5'-Short tail ] to the sequence 5'-GCGGGC-3'. These rich GC tails produce amplicons that allow easier identification by melting curve or agarose electrophoresis. | | | |
| --- | --- | --- | --- |
|  |  |  |  |
| Marker Name | Vector Base ID | SNP position | Primer sequence  Allele specific primer #1  Allele specific primer #2  Reverse primer |
| **Chromosome 1** |  |  |  |
| *CathepB* | AAEL007599 | 231 | [longtail]-GATGTCCTGTGGGTTGACCAGACTG |
|  |  |  | [short tail]-GATGTCCTGTGGGTTGACCAGATTA |
|  |  |  | GCCTACTTCAAAGGTGTTYTGT |
|  |  |  |  |
| *AChE-2* | AAEL012141 | 381 | [longtail]-GTGACCCAGACCTAATAACCGTG |
|  |  |  | [short tail]-GTGACCCAGACCTAATAACCATA |
|  |  |  | GCGTSACMGGWGACAGTAGATG |
|  |  |  |  |
| *CCEae1C* | AAEL003195 | 828 | [longtail]-TACTCCTATTGCAGATTTTATTCAC |
|  |  |  | [short tail]-TACTCCTATTGCAGATTTTATTTAT |
|  |  |  | TCCACGAGGAATCGGKAT |
|  |  |  |  |
| *CCEae2C* | AAEL003196 | 1209 | [longtail]-TGTTATGGTAGAATGGACTCAAGTC |
|  |  |  | [short tail]-TGTTATGGTAGAATGGACTCAAATT |
|  |  |  | CAGTTAGAACCACCGCTTT |
|  |  |  |  |
| *CCEae5C* | AAEL003201 | 411 | [longtail]-TACATTTTCTTGGATGAGATGAGCA |
|  |  |  | [short tail]-TACATTTTCTTGGATGAGATGAACT |
|  |  |  | CACGGCGGATCMTTTACC |
|  |  |  |  |
| *Chitan-1* | AAEL002972 | 66 | [longtail]-GATACTGATGACGGCGGTGGTGGTA |
|  |  |  | [short tail]-GATACTGATGACGGCGGTGGTGATT |
|  |  |  | CGGCTGTTGACCTTGTGCTTCC |
|  |  |  |  |
| *Aegi22* | AAEL009932 | 3864 (utr) | [longtail]-TCTTTGTGAAATGTTCGCATCGAGC |
|  |  |  | [short tail]-TCTTTGTGAAATGTTCGCATCGGGT |
|  |  |  | CCTTYMATTGGAGTTCCCT |
| **Chromosome 2** |  |  |  |
| *CCEbe20** | AAEL012509 | 936 | [long tail]-ACGGATGATTAGCCAGGTAT |
|  |  |  | [short tail]-ACGGATGATTAGCCAGGCAA |
|  |  |  | TACAAYCCATTCTCACCGC |
|  |  |  |  |
| *InsRec* | AAEL002317 | 2547 | [longtail]-GACCCGTATCTGGCGCTGGGTGATG |
|  |  |  | [short tail]-GACCCGTATCTGGCGCTGGGTGGTA |
|  |  |  | GAAGAAGGAAGGTGCYGA |
|  |  |  |  |
| *Fxa* | AAEL007420 | 313 | [longtail]-TCCGAAGGGCAAATTACCAGAWATG |
|  |  |  | [short tail]-TCCGAAGGGCAAATTACCAGAWGTA |
|  |  |  | GCTCTAACGCTGTCCACT |
|  |  |  |  |
| *CYP9J32** | AAEL008846 | 780 | AAGTTTGATGATTAAGATGGG |
|  |  |  | [longtail]-ACTGCTTCCTTGATGATTGTG |
|  |  |  | [short tail]-ACTGCTTCCTTGATGATTATT |
|  |  |  |  |
| *Arc4* | AAEL000723 | 2331 | [longtail]-CAGTCAGTACAACACGGACACGTAG |
|  |  |  | [short tail]-CAGTCAGTACAACACGGACACGCAA |
|  |  |  | CATTTCCTTCTTGTGCWTCTGC |
|  |  |  |  |
| *CarboxA* | AAEL010782 | 357 (orf) | [longtail]-AGGTTCACAGCAGGTATTCTAAATG |
|  |  |  | [short tail]-AGGTTCACAGCAGGTATTCTAAGTT |
|  |  |  | ATTCTGCCCTGTTMTCCA |
|  |  |  |  |
| *DDC* | AAEL014238 | 853 | [longtail]-TCAATCTCACTCATCTGTAGAAGGA |
|  |  |  | [short tail]-TCAATCTCACTCATCTGTAGAAAGC |
|  |  |  | AACTCCTCCAAGCAAACC |
|  |  |  |  |
| *LF357* | AAEL002081 | 168 | [longtail]-TTGTTTGGTAGGTTGATGATCGGAG |
|  |  |  | [short tail]-TTGTTTGGTAGGTTGATGATCGAAC |
|  |  |  | TGACCAAGCAACCCAAGA |
|  |  |  |  |
| *sin3J* | AAEL014711 | 4011 | [longtail]-ATCCAGCGTAAATGCAATGTAGTCG |
|  |  |  | [short tail]-ATCCAGCGTAAATGCAATGTAGCCA |
|  |  |  | GACGGTAACATGGAGGCG |
| **Chromosome 3** |  |  |  |
| *CCEae2D** | AAEL010389 | 807 | [long tail]-TTCACRTTTTCCGTTCGTCAA |
|  |  |  | [short tail]-TTCACRTTTTCCGTTCGTTAG |
|  |  |  | AAAGCCACCCCAGAAGATA |
|  |  |  |  |
| *LF396* | DQ440442.1 | 346 | [longtail]-GAACGCAATCCATRGTATCGTCGGC |
|  |  |  | [short tail]-GAACGCAATCCATRGTATCGTCAGG |
|  |  |  | TCCGAGAAGCCAAACCAC |
|  |  |  |  |
| *hsp70* | DQ440299.1 | 414 | [longtail]-CGCCGAAGCCTACCTGGGCAAGTCG |
|  |  |  | [short tail]-CGCCGAAGCCTACCTGGGCAAGCCA |
|  |  |  | AAGTAAGCAGGCACTATGTC |
|  |  |  |  |
| *vitg* | AAEL010434 | 5066 | [longtail]-CGAAGCGTTGTAGTAATCGCCRGAG |
|  |  |  | [short tail]-CGAAGCGTTGTAGTAATCGCCRAAA |
|  |  |  | TGGAAAGTCMAACTACATCAA |
|  |  |  |  |
| *para** | AAEL006019 | 2336 | [longtail]-ACAAATTGTTTCCCACCCGCACCGG |
|  |  |  | [short tail]-ACAAATTGTTTCCCACCCGCACTGA |
|  |  |  | TGATGAACCSGAATTGGACAAAAGC |
|  |  |  |  |
| *Apyr1* | AAEL006347 | 447 | [longtail]-ATCGGGGTCGTTGTTCATTTCC |
|  |  |  | [short tail]-ATCGGGGTCGTTGTTCATTCCT |
|  |  |  | TGGCAGAACTGAATAAAGAG |
|  |  |  |  |
| AChE-1 | EF209048 | 34 | [longtail]-GCCTAATAACCCGATTGCAAG |
|  |  |  | [short tail]-GCCTAATAACCCGATTGCGAA |
|  |  |  | TTCCCAAACTGCACAGTATCA |
