# Supplementary material for: QTL Mapping of Genome Regions Controlling Temephos Resistance in Larvae of the Mosquito Aedes aegypti
Source: PLoS Negl Trop Dis. 2014 Oct 16;8(10):e3177. doi: 10.1371/journal.pntd.0003177 (PMC4199591; doi:10.1371/journal.pntd.0003177)
Supplement: Table S2 — Names and locations of markers used in mapping of temephos resistance QTL in Aedes aegypti. (DOCX) [file pntd.0003177.s002.docx]

**Table S2.**

Chromosome Linkage Physical Segregation in Publication

Locus GenBank # (cM) division SLDxIq IqxSLD

| **Chromosome 1** |  |  |  |  |  |  |
| --- | --- | --- | --- | --- | --- | --- |
| CathepB | L41940 | 0.0 | 1p3.4 | + | + | [1] |
| 176TG1 | GF101910 | 0.3 | 1p3.4 | + | + | [2] |
| 12ACG1 | GF101911 | 18.7 | 1p2.3 | + | + | [2] |
| 12ATG1 | GF101912 | 18.8 | 1p2.3 |  | + | [2] |
| 12CGT1 | GF101950 | 19.1 | 1p2.3 | + | + | [2] |
| 71CGT1 | GF101913 | 26.9 | 1p1.4 | + | + | [2] |
| 71AT1 | GF101914 | 27.2 | 1p1.4 |  | + | [2] |
| 192TAAA1 | GF101916 | 29.6 | 1p1.3 | + | + | [2] |
| 335CGA1 | GF101917 | 40.4 | 1q1.2 | + |  | [2] |
| AChE-2 | AAEL012141 | 40.7 | 1q1.2 | + |  | Current MS |
| CCEae1C | AAEL003195 | 48.5 | 1q2.1 | + | + | Current MS |
| CCEae2C | AAEL003196 | 48.7 | 1q2.1 | + | + | Current MS |
| CCEae5C | AAEL003201 | 49.0 | 1q2.1 | + | + | Current MS |
| 88AT1 | DV389063 | 56.3 | 1q3.2 | + | + | [3] |
| 88GAA1 | GF101919 | 56.5 | 1q3.2 | + | + | [2] |
| Chitan1 | AF026491 | 56.8 | 1q4.1 | + |  | [1] |
| 68ATGG1 | GF101921 | 69.6 | 1q4.4 |  | + | [2] |
| 440AAC1 | GF101923 | 69.7 | 1q4.4 | + |  | [2] |
| 440TGTA1 | GF101925 | 69.7 | 1q4.4 |  | + | [2] |
| AEGI22 | BI099650 | 70.0 | 1q4.4 | + |  | [4] |
|  |  |  |  | 16 | 15 |  |
| **Chromosome 2** |  |  |  |  |  |  |
| 145TAAA1 | GF101926 | 0.0 | 2p4.4 | + |  | [2] |
| 328CTT1 | GF101929 | 5.8 | 2p4.2 |  | + | [2] |
| CCEbe20 | AAEL012509 | 9.7 | 2p3.4 |  | + | [5] |
| insrecp | U72939 | 13.6 | 2p3.2 |  | + | [1] |
| fxa | AF050133 | 21.4 | 2p2.5 |  | + | [4] |
| 462GA1 | DU169902 | 29.2 | 2p2.1 | + | + | [7] |
| CYP9J32 | AAEL008846 | 36.9 | 2q1.1 |  | + | [5] |
| Arc4 | BH214543 | 40.8 | 2q1.3 | + | + | [4] |
| Carbox | AF165923 | 46.7 | 2q2.3 | + | + | [1] |
| 109CT1 | DV362806 | 48.6 | 2q2.4 | + | + | [3] |
| DDC | AI638914 | 50.6 | 2q3.1 |  | + | [1] |
| LF357 | BM005495 | 54.4 | 2q3.3 |  | + | [4] |
| 25AAG1 | GF101927 | 62.2 | 2q3.7 | + |  | [2] |
| 121GA1 | GF101928 | 66.1 | 2q4.2 | + | + | [2] |
| 1132CT1 | GF101930 | 69.8 | 2q4.4 | + | + | [2] |
| Sin3J | AI561370 | 70.0 | 2q4.4 | + | + | [1] |
|  |  |  |  | 9 | 14 |  |
| **Chromosome 3** |  |  |  |  |  |  |
| 301ACG1 | GF101931 | 0.0 | 3p4.4 | + | + | [2] |
| CCEae2D | AAEL015578 | 6.1 | 3p4.2 |  | + | [6] |
| LF396 | BM005498 | 10.2 | 3p3.4 | + |  | [4] |
| hsp70 | AI658418 | 18.3 | 3p2.3 | + | + | [4] |
| 766ATT1 | GF101934 | 32.5 | 3q1.1 | + |  | [2] |
| 69TGA1 | GF101935 | 34.5 | 3q1.2 | + | + | [2] |
| vitg | L41842 | 42.7 | 3q2.2 |  | + | [1] |
| LF261 | BM378052 | 44.7 | 3q2.3 | + | + | [4] |
| para | AAEL006019 | 48.8 | 3q2.5 | + |  | [6] |
| 86AC1 | DV309356 | 56.9 | 3q3.4 | + | + | [3] |
| 201TTA1 | GF101936 | 64.6 | 3q4.4 |  | + | [2] |
| 470CT2 | GF101938 | 64.8 | 3q4.4 | + | + | [2] |
| APYR1 | L12389 | 65.0 | 3q4.4 |  | + | [4] |
| AChE-1 | EF209048 | 65.0 | Unknown |  | + | [4] |
|  |  | SC1.9 |  | + | + | Current MS |
|  |  |  |  | 10 | 12 |  |
|  |  |  |  |  |  |  |

1. Fulton RE, Salasek ML, DuTeau NM, Black WC (2001) SSCP analysis of cDNA markers provides a dense linkage map of the Aedes aegypti genome. Genetics 158: 715-726.

2. Lovin DD, Washington KO, deBruyn B, Hemme RR, Mori A, et al. (2009) Genome-based polymorphic microsatellite development and validation in the mosquito Aedes aegypti and application to population genetics in Haiti. Bmc Genomics 10.

3. Slotman MA, Kelly NB, Harrington LC, Kitthawee S, Jones JW, et al. (2007) Polymorphic microsatellite markers for studies of Aedes aegypti (Diptera : Culicidae), the vector of dengue and yellow fever. Molecular Ecology Notes 7: 168-171.

4. Gomez-Machorro C, Bennett KE, Munoz MD, Black WC (2004) Quantitative trait loci affecting dengue midgut infection barriers in an advanced intercross line of Aedes aegypti. Insect Molecular Biology 13: 637-648.

5. Saavedra-Rodriguez K, Suarez AF, Salas IF, Strode C, Ranson H, et al. (2012) Transcription of detoxification genes after permethrin selection in the mosquito Aedes aegypti. Insect Molecular Biology 21: 61-77.

6. Saavedra-Rodriguez K, Urdaneta-Marquez L, Rajatileka S, Moulton M, Flores AE, et al. (2007) A mutation in the voltage-gated sodium channel gene associated with pyrethroid resistance in Latin American Aedes aegypti. Insect Molecular Biology 16: 785-798.

7. Chambers EW, Meece JK, McGowan JA, Lovin DD, Hemme RR, et al. (2007) Microsatellite isolation and linkage group identification in the yellow fever mosquito Aedes aegypti. Journal of Heredity 98: 202-210.
